# Membranes Based on Polyvinylidene Fluoride and Radiation-Grafted Sulfonated Polystyrene and Their Performance in Proton-Exchange Membrane Fuel Cells

**DOI:** 10.3390/polym14183833

**Published:** 2022-09-14

**Authors:** Daniil V. Golubenko, Oleg V. Korchagin, Daria Yu. Voropaeva, Vera A. Bogdanovskaya, Andrey B. Yaroslavtsev

**Affiliations:** 1 Kurnakov Institute of General and Inorganic Chemistry RAS, 31 Leninsky Avenue, 119991 Moscow, Russia; 2 Frumkin Institute of Physical Chemistry and Electrochemistry RAS, 31 Leninsky Avenue, 119071 Moscow, Russia

**Keywords:** proton conducting membranes, radiation-induced graft copolymerization, polystyrene sulfonate, polyvinylidene fluoride, proton-exchange membrane fuel cells

## Abstract

Proton-exchange membranes based on gamma-irradiated films of PVDF and radiation-grafted sulfonated polystyrene with an ion-exchange capacity of 1.8 meq/g and crosslinking degrees of 0 and 3% were synthesized. A solvent-free, environmentally friendly method of styrene grafting from its aqueous emulsion, with a styrene content of only 5 vol.% was used. Energy dispersive X-ray mapping analysis showed that the grafted sulfonated polystyrene is uniformly distributed throughout the membrane thickness. The obtained materials had a proton conductivity up to 132 mS/cm at 80 °C and a hydrogen permeability of up to 5.2 cm^2^/s at 30 °C, which significantly exceeded similar values for Nafion^®^-212 membranes. The resulting membranes exhibited a H_2_/O_2_ fuel cell peak power density of up to 0.4 W/cm^2^ at 65 °C. Accelerated stability tests showed that adding a crosslinking agent could significantly increase the stability of the membranes in the fuel cells. The thermal properties and crystallinity of the membranes were investigated through differential scanning calorimetry and X-ray powder diffraction methods. The conductivity, water uptake, and mechanical properties of the membranes (stress–strain curves) were also characterized.

## 1. Introduction

The efforts of humanity to improve the environment have a significant impact on the modern energy industry. The main trends are energy conservation, decarbonization, and transition to renewable energy sources [1,2,3,4]. Great hopes have been placed on hydrogen energy. H_2_/O_2_ fuel cells can serve as environmentally friendly energy sources, the final product of which is only water [5,6]. Equally important, the hydrogen cycle can provide a stable energy supply for solar panels and wind turbines, the operation of which is stochastic [7,8,9]. This cycle involves the production of hydrogen via water electrolysis using renewable energy sources, followed by the generation of energy from the received hydrogen in the absence of wind or during the nighttime [10]. While lithium-ion batteries can cover daily fluctuations in power supply, the hydrogen cycle is essential for protecting seasonal changes in power supply, as the batteries lose energy through self-discharge [11].

However, despite the great focus towards this field, hydrogen power has not yet reached the proper level of progress. The reason for this is the high cost of the most common proton-exchange membrane fuel cells (PEMFC). This, in turn, is determined by the high price of platinum-based catalysts and perfluorinated Nafion^®^ or Aquivion^®^ membranes [4]. Precisely because of their high cost, perfluorinated membranes cannot compete in such water treatment technology as electrodialysis with less selective, but much cheaper, heterogeneous membranes [6,12,13]. However, for PEMFC applications, the high selectivity and stability of homogeneous perfluorinated membranes are crucial [4]. Thus, it is necessary to develop cheaper membranes with a high selectivity to promote hydrogen energy.

The reason for the low selectivity of heterogeneous ion-exchange membranes is the presence in their structure of large 1 μm pores, which are formed in the stage of hot pressing or rolling the particles of an ion-exchange resin and plasticizer [14,15,16]. One of the most promising ways to solve this problem would be radiation-induced graft copolymerization [17,18,19,20,21]. In this method, the grafting polymer is formed directly in a base polymer matrix by spreading the chains, thus creating a space to grow without forming macropores [22]. The radiation-grafted ion-exchange membranes combine high ionic conductivity and selectivity. Previously, we successfully obtained cations and anions conducting graft copolymers based on UV-irradiated polymethylpentene and functionalized polystyrenes [23,24,25]. At the same time, polymethylpentene cannot ensure proper oxidative stability in the fuel cell environment due to the presence of tertiary carbon atoms in its structure, which have an increased reactivity.

To create more stable membranes, it is necessary to use fluorinated base polymers. Unfortunately, Teflon is not suited for this purpose because of its high crystallinity. A possible compromise is to use a polyvinylidene fluoride (PVDF), where half of the hydrogen atoms are replaced by fluorine. This polymer has also attracted the attention of researchers as a matrix for producing membranes through graft copolymerization. However, most studies have been devoted to membranes for filtration processes [26,27,28,29]. The synthesis of grafted ion-exchange membranes based on PVDF films has received considerably less attention. For example, Sezgin et al. [30] described the process of PVDF-based membrane synthesis by grafting poly(1-vinyl-1,2,4-triazole) doped with triflic acid. The maximum proton conductivity of 6 mS/cm at 150 °C was achieved for membranes with a degree of grafting of 21%. Lepit et al. achieved a proton conductivity of 1 mS/cm at 100 °C for PVDF membranes with grafted 1-vinylimidazole prepared via radiation-induced graft polymerization [31].

α-methylstyrene and acrylonitrile were grafted after the treatment of PVDF with NaOH and Na_4_SiO_4_ by Li et al. [32]. Membranes with an ion-exchange capacity of 0.41–0.89 meq/g and a proton conductivity significantly lower than that of Nafion^®^ were obtained. In addition, they were characterized by a low strength due to cracking of the polymer matrix. Styrene grafted PVDF proton-exchange membranes for vanadium redox flow battery with a power density of 336 mW/cm^2^ have been described [33]. These membranes were produced according to the procedure described in [34]. The ion-exchange capacity of these membranes reached 2.67 meq/g, and their ionic conductivity and selectivity were slightly lower than for Nafion^®^ [33].

The pore-filling method is promising for making selective membranes [35,36,37,38]. We recently used the pore-filling method and its combination with radiation-induced grafting to synthesize sulfonated polystyrene in track-etched membranes based on the PVDF matrix [39]. Both approaches yielded membranes that surpassed Nafion^®^ in proton conductivity, but they also had a good enough gas permeability. The high ion-exchange capacity of polystyrene sulfonate and the optimal geometry of the tracks filled with it, located perpendicular to the surface, can be considered to be advantages of these approaches. It can be assumed that the reason for the higher gas permeability is incomplete pore filling due to non-ideal styrene filling, which is difficult to control or is due to shrinkage of the polystyrene during polymerization.

As the synthesis of radiation-grafted membranes is accompanied by a high consumption of organic solvents, the solvent-free method has recently attracted attention [40,41,42]. Most often, it is used for the grafting of expensive and toxic chloromethylstyrene. The approach uses an emulsion of monomer in water as a reaction mixture with a relatively low monomer content of 3–7 vol.%, instead of traditional organic solvent/monomer mixtures with a monomer content of 10–80%. To the best of our knowledge, this approach has not previously been used to produce cation-exchange membranes based on grafted polystyrene.

Thus, this work is aimed to obtain an ion-exchange membrane based on PVDF films with radiation-grafted polystyrene sulfonate, as well as to study its transport properties and the possibility of its use in a H_2_/O_2_ PEMFC.

## 2. Materials

The following reagents were used: 50 µm PVDF films (GoodFelow, London, UK), styrene (99%, extra pure, stabilized, Acros Organics), isopropyl alcohol (chemically pure, Chimmed), argon (99,993%, Air Liquide), 1,2-dichloroethane (chemically pure, Chimmed), sulfurochloridic acid (99%, Sigma-Aldrich, St. Louis, MO, USA), HCl, NaCl, NaOH, and anhydrous CaCl_2_ (chemically pure, Chimmed).

The PVDF films were irradiated using a Cs-137 gamma source (source activity 1.8 Gy/min) until a dose of 20 kGy was collected. After irradiation, the film was stored at −18 °C to decrease the radical deactivation rate. Styrene was purified from the inhibitor using the following method. First, styrene was stirred with sodium hydroxide for 24 h and then washed with distilled water three-fold. Then, it was dried over anhydrous calcium chloride for 24 h and distilled at 70 °C under a vacuum at a residual pressure of 60 mm Hg. The vacuum was generated using a vacuum pump PC 3001 VARIO PRO (Vacuumbrand, Germany) with a pressure controller CVC 3000. Divinylbenzene was purified by passing through a column filled with a sorbent to remove 4-tert-butylcatechol (Sigma-Aldrich).

## 3. Membrane Synthesis

The method of grafting styrene onto the pre-irradiated film was based on the procedure described in [43]. Grafting was carried out in a monomer reaction mixture at 95 °C in an argon atmosphere with stirring in a tailor-made metal reactor. The key difference was the use of dispersion of 5 vol.% of styrene (or its mixture with divinylbenzene) in water with dodecylbenzene sulfonate as an emulsion stabilizer as a monomer mixture. Such a modification significantly reduced the monomer consumption and increased the environmental compatibility of the process. After grafting, the films were washed with dichloromethane and water and were dried in the air. The grafting degree (*GD*, *%*) of polystyrene was calculated by the following equation:

$$GD=\frac{m_{1}-m_{0}}{m_{0}}\cdot 100\%$$

where *m*_1_ and *m*_0_ were weights of the film samples after and before styrene grafting.

Sulfonation of the obtained composites was carried out in a 2 vol.% chlorosulfuric acid solution in 1,2-dichloroethane for 3 h. After sulfonation, the films were washed in isopropyl alcohol, dried in the air, and hydrated with distilled water at 80–90 °C for 3–4 h.

## 4. Methods

The ionic conductivity (*σ, mS/cm*) of the membranes in contact with deionized water was measured by impedance spectroscopy using a potentiostat-galvanostat with a P-40 X impedance measurement module (Elins, Russia) in a two-electrode symmetric cell (Cu current collector/carbon foil/membrane/carbon foil/Cu current collector). The temperature control was performed using a MR Hei-Standard heating magnetic stirrer (Heidolph, Germany).

The water uptake (*WU*, *%*) of the membranes in contact with deionized water was determined by the mass loss of the membrane after drying the sample in an ES-4610 oven (Ecohim, Russia) at 80 °C. A detailed description of the methodology is given in the work of [43]. To determine the water uptake at a constant relative humidity (RH) and room temperature, the membrane sample was weighed after keeping it in a desiccator over a saturated solution of appropriate salt for 3 days.

The ion-exchange capacity (*IEC*, meq/g) was determined by acid–base titration using a pH meter (Econix, Russia) according to the procedure described in [44], and was calculated per mass of the sample dried at 80 °C.

Membrane hydrogen permeability (*P*, cm^2^/s) was measured in a two-section cell by the flow of wetted hydrogen according to the procedure described in [44]. The samples were equilibrated with deionized water before determining the gas permeability. The outlet hydrogen concentration was determined with a Kristallux-4000 M chromatograph (Meta-Chrom, Russia) equipped with a thermal conductivity detector (30 mA current) and a packed column (Mole Seive 5 A sorbent, 2 m, 30 °C, 20 cm^3^/min, Ar).

Hydration numbers (*λ*, H_2_O/-SO_3_H) were calculated based on the water uptake and ion-exchange capacity using the equation given in [44].

The mechanical properties were studied using an H5KT tensile testing machine (Tinius Olsen, Horsham, PA, USA). Before measurement, the specimens were air-conditioned (24 °C, 25% relative humidity) for 24 h. Rectangular specimens with a length of 80 mm (base length 50 mm) and a width of 10 mm were used. The tensile rate was 5 mm/min. Three samples were examined for each type of membrane. A detailed description is given in [45].

Differential scanning calorimetry was performed on a STA 449F1 (Netzsch, Germany) in aluminum crucibles under a helium flow rate of 20 mL/min. The sample was analyzed in three steps, including heating from 0 to 200 °C, cooling from 200 to 0 °C, and again heating to 200 °C at a heating/cooling rate of 10 °C/min. To calculate enthalpies of melting, the apparatus was calibrated using a standard tin sample.

The IR spectra of the films were collected using a Nicolet iS5 FTIR spectrometer (Thermo Fisher Scientific, Waltham, MA, USA) with a Quest Specac attachment with a diamond crystal (spectral range 500–4000 cm^−1^, 32 scans, resolution 2 cm^−1^) in the attenuated total reflection mode. The OMNIC^®^ IR software Advanced ATR correction was used to correct the resulting spectra (bounces number—of 1, angle of incidence of 45°, and refractive index of 1.5).

### 4.1. Performance of Fuel Cell

#### 4.1.1. Technique for the Formation of Membrane Electrode Assemblies

Membrane electrode assemblies (MEAs) were formed by applying catalytic ink to the membrane using an airbrush. The area of the electrodes was 5 cm^2^. Catalytic ink was prepared by ultrasonic treatment of an aqueous-alcohol catalyst suspension with the addition of a Nafion^®^ ionomer solution. Then, 40 wt.% Pt/carbon nanotubes (cathode of 0.6 mgPt/cm^2^, anode of 0.3 mgPt/cm^2^) was used as a catalyst for the cathode and anode. This catalyst’s synthesis technique and characteristics were described earlier [46]. The mass ratio of the ionomer/carbon material in ink was ~0.9, calculated on a pure ionomer without a solvent. The ink was applied at a temperature of 80–90 °C. The formed electrodes were dried in a vacuum oven and clamped in an ElectroChem test cell between two gas diffusion layers (GDL) of 39 BB. The degree of compression of MEAs (20–25%) was regulated by matching the thickness of the Teflon spacers.

#### 4.1.2. Testing of MEAs

The testing of MEAs in the test cell was performed on a specialized ElectroChem fuel cell test station (ElectroChem, Woburn, MA, USA). Electrochemical measurements were performed using an Elins P-45x potentiostat-galvanostat with an FRA module.

The voltammetry curves of H_2_/O_2_ PEMFC with the studied MEAs were recorded under steady-state conditions. The measurements were performed using hydrogen and oxygen without overpressure, with 100% RH of the gases at a cell temperature of 65 °C. The electrochemically active surface area of the platinum catalyst (S_Pt_) in the MEAs cathode was determined based on cyclic voltammetry recorded at a rate of 50 mV/s.

The stability of MEAs was assessed using the accelerated stress test protocol [47]. During the trials, oxygen and hydrogen with 30% RH were fed without overpressure into the fuel cell heated to 90 °C. The MEAs were kept under these conditions at an open-circuit voltage (OCV). Before and after the stability tests, the S_Pt_ of the cathode and the high-frequency resistance of the MEA were measured [48]. Hydrogen crossover was also evaluated as a current established at a voltage of 0.4 V in an inert atmosphere at the cathode side when hydrogen passed through the anode.

## 5. Results and Discussions

### 5.1. Membranes Characterization

**Figure 1:**
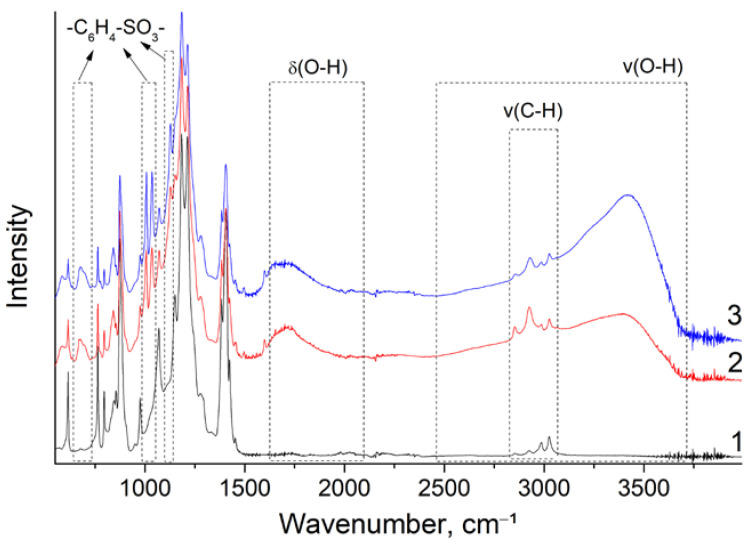
FTIR spectra of the initial PVDF film (1) and the obtained MCG-3 (2) and MCG-0 (3) membranes in proton form dried under air with an RH of 20–30%. The radiation-grafted membranes studied were designated as MCG-X, where X is the crosslinking degree equal to the divinylbenzene volume fraction in the styrene-divinylbenzene mixture.

To understand the processes occurring during the synthesis of membranes, the changes in the IR spectra were analyzed. For the radiation-grafted membranes, new peaks with maximums at 574, 674, 1000, 1127, and 1600 cm^−1^ were observed (Figure 1), which corresponded to vibrations of the polystyrene sulfonic acid [49]. This confirmed the grafting and sulfonation of polystyrene. In addition, because of the hydration of sulfonic groups, the peaks of deformation and the stretching vibrations of water with maximums at 1650–1680 and 3390–3400 cm^−1^ appeared. The latter appeared as a broad halo extending into the region of low frequencies, typical for systems with strong hydrogen bonds in hydrate shells of hydrated protons. Finally, it is worth noting that the fraction of weakly bound water was higher for the non-crosslinked membrane, as evidenced by the higher intensity of the 3400 cm^−1^ peak [50].

The thickness of radiation-grafted membranes was more than the initial PVDF film (Table 1). As the properties of the membranes largely depend on their thickness, Nafion^®^-212, with a thickness of 51 µm, was chosen as a reference sample. Both crosslinked and non-crosslinked samples have a high IEC, which is more than twice as high as that of Nafion^®^ membranes. Notably, the IEC values for the obtained samples were almost the same, indicating that crosslinking did not practically limit the availability of functional groups and did not eliminate their participation in the transport processes.

**Figure 2:**
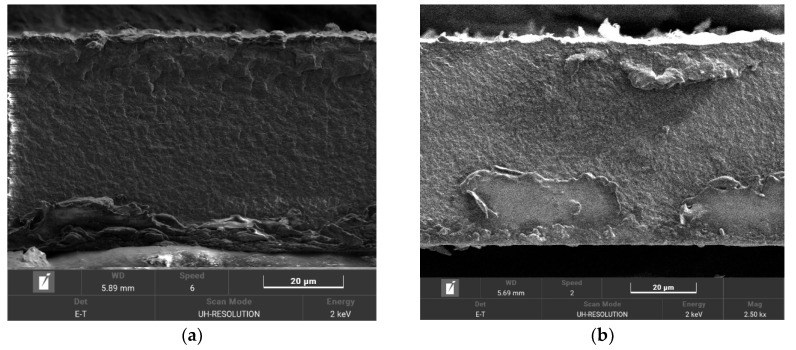

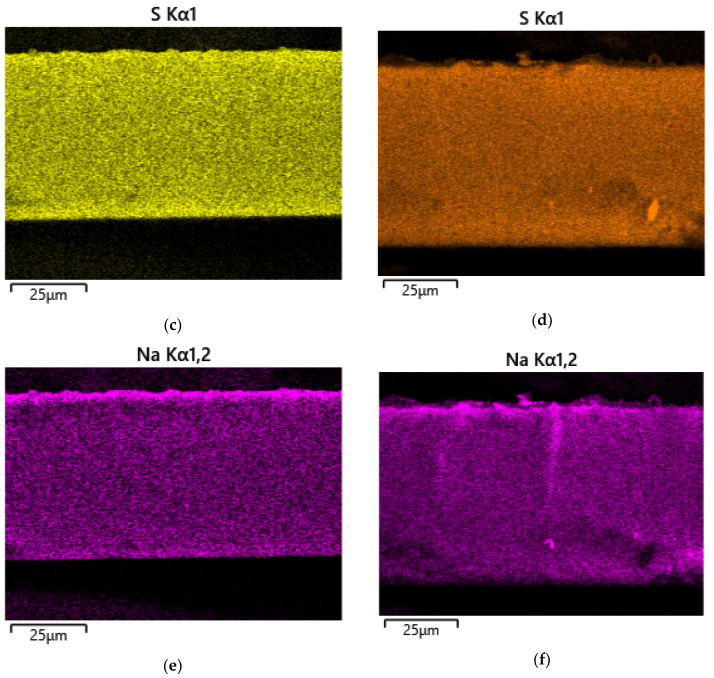
SEM micrographs of the cross-section of MCG-0 (**a**) and MCG-3 (**b**) membrane, as well as distribution of S (**c**,**d**) and Na (**e**,**f**).

According to the micrographs, the obtained films were homogeneous (Figure 2a,b). The formation of a separate polystyrene sulfonate phase was observed not on the surface or in the volume. At the same time, no defects or inhomogeneity of sulfur and sodium distributions could be seen on the micrographs because the size of the phase-separation inhomogeneities characterized for graft-copolymers was smaller than the resolution of the electron microscope. In addition, the distribution of sulfur and sodium over the membrane thickness was also homogeneous, indicating that the grafting and further sulfonation proceed smoothly over the entire thickness (Figure 2c,f and Figure S1).

The value of ionic conductivity was determined not only by the charge carrier concentration, but also by the water uptake of the membranes. Water molecules are not solely involved in the Grotthuss transport mechanism, but also determine the size of the pores and channels, which limits membranes’ conductivity [51]. The hydration numbers of the obtained membranes were close to that of the Nafion^®^-212 membranes, but were somewhat lower for the crosslinked ones.

**Figure 3:**
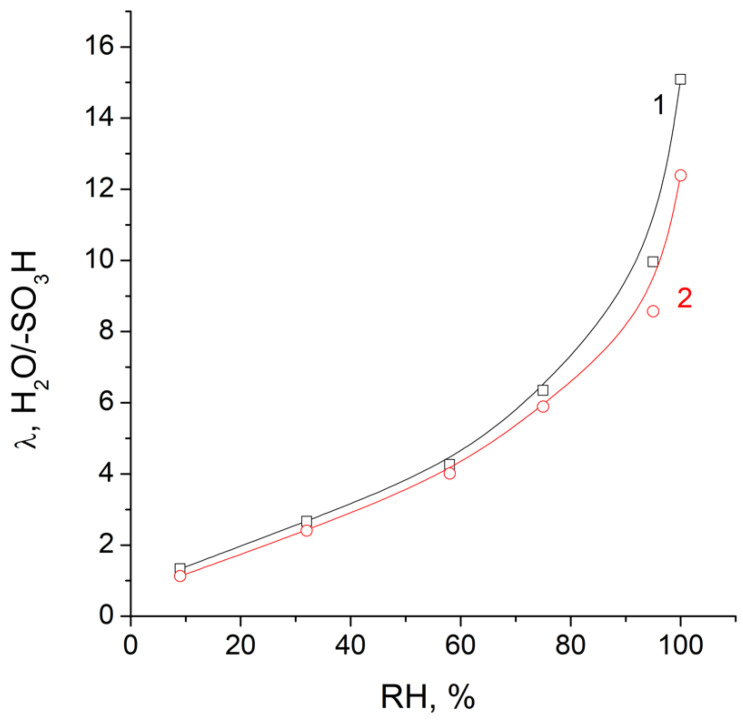
Dependence of the hydration numbers of MCG-0 (1) and MCG-3 (2) on the relative humidity (RH) at room temperature (100% humidity denotes the contact with water).

The dependence of water uptake on the relative humidity was also important for the membranes used in PEMFC. The rapid decrease in water uptake made it necessary to maintain a high, close to 100% humidity for the feed gases in the fuel cells, and significantly complicated their design. The dependencies of the degree of hydration of the obtained membranes on the relative humidity, shown in Figure 3, were similar to those for other membranes with sulfonic groups. Crosslinking reduced the hydration numbers at all relative humidities.

**Figure 4:**
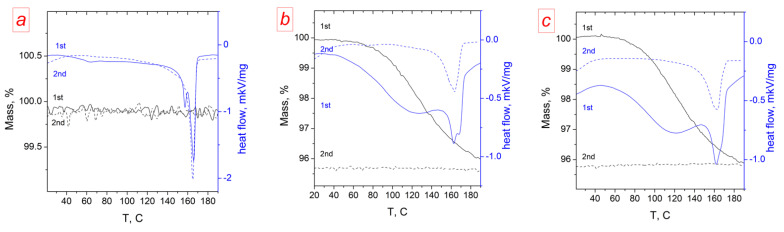
DSC curves of three-stage heating–cooling–heating analysis for the studied samples: (**a**) PVDF, (**b**) MCG-0, and (**c**) MCG-3. The curves for the first and second heating are shown.

The DSC curve of the MCG-3 membrane indicates that during first heating, a complex process occurred, with at least two endothermic effects (Figure 4b). The first one was broad and was accompanied by mass loss due to dehydration. The second narrower one corresponded to the melting of the PVDF crystalline phase [52]. This relationship was confirmed by the fact that during cooling and second heating, the first of them disappeared, and there was no change in the mass of the sample. It is also indicative that for the initial PVDF film, the first endothermic effect and the corresponding mass loss were absent (Figure 4a). This makes it possible to determine the value of the endothermic effect of the rearrangement of the crystallized phase of the sample and to estimate the degree of crystallinity α_mem_ (Table 2). The data obtained showed that the crystallization heat and crystallinity degree for both radiation-grafted membranes were close and approximately half that for the original PVDF. The decrease in the degree of crystallinity of PVDF in the radiation-grafted membranes relative to the original base film was also confirmed by XRD. The intensity of reflections corresponding to the crystalline phase of PVDF was reduced after grafting (Figure S2).

The decrease in the degree of crystallinity for radiation-grafted ion-exchange membranes was associated with two phenomena—an increase in sample mass due to grafting of the amorphous polymer (dilution effect) and amorphization of the base polymer [53,54]. Therefore, to estimate the degree of crystallinity of the base polymer (α_PVDF_), it is necessary to consider the PVDF content in the final membrane, which can be done based on the degree of polystyrene grafting and ion-exchange capacity using the following equation:

$$\alpha_{PVDF}=\alpha_{mem}/\left(\left[(1-IEC\cdot M\left({\mathrm{SO}}_{3}\right)\right]\cdot \frac{100}{\left(100-DG\right)}\right)$$

where *M*(SO_3_) is the molar weight of SO_3_ in g/mol.

For the obtained membranes, a decrease in the degree of crystallinity of the base polymer (PVDF) was also observed (Table 2). Such amorphization occurred after the sulfonation/hydration process. This is explained by the distortion of part of the base polymer crystallites by the internal stress arising during the hydration of sulfonated polystyrene and the significant increase in the membrane volume [53].

The high ion-exchange capacity, water uptake, and low degree of crystallinity created favorable conditions for proton transport. Indeed, the proton conductivity of the membranes obtained at room temperature was several times higher than that of Nafion^®^-212. This difference only increased with the increase in temperature and reached 132 mS/cm at 80 °C for MCG-3, approaching the highest values for the proton conductivity of solid electrolytes. At the same time, despite the relative carrier concentration and degree of crystallinity, the conductivity of crosslinked membranes was one and a half times lower than that of the non-crosslinked membrane. Similar to Nafion^®^ membranes, the MCG ionic conductivity decreased sharply with the decrease in humidity (Table 3). However, the difference in conductivity between crosslinked and non-crosslinked membranes was evened out. Thus, at RH = 30%, the conductivity of both membranes was practically equalized.

At the same time, an essential factor for fuel cell proton conducting membranes is gas permeability, which determines the crossover of feed gases through the membrane, which does not lead to electricity generation. Its ratio to proton conductivity determines the selectivity of membranes in fuel cells. It is known that an increase in ionic conductivity with an increase in water uptake usually leads to a decrease in the selectivity of ion-exchange membranes [25,55,56,57]. It is explained by the rise in the fraction of electroneutral solution in the center of the pores, which determines the non-selective transport [51]. However, the gas permeability of the obtained membranes is also low. This parameter also surpassed Nafion^®^-212 by three to four times. It is also worth noting that, together with a decrease in water uptake, crosslinking led to a reduction in hydrogen permeability of the obtained membranes by 36% (Table 1). A comparison of the gas permeability of the obtained membranes with PVDF and Nafion^®^-212 indicated that the dominant part of the gas flowed through the hydrophilic part of the membranes containing bulk backbones, which prevented the ordered packing of chains. Their absence in PVDF films determined their much lower gas permeability.

**Figure 5:**
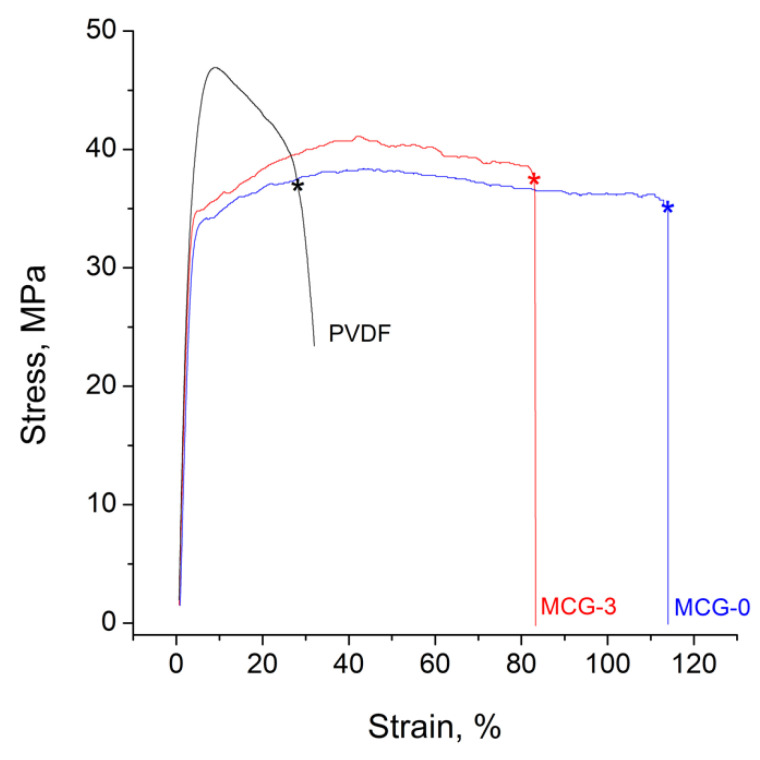
Stress–strain curves of the obtained membranes and the initial PVDF film. One of three curves for each sample type is given. The breakpoint is marked with a star sign.

Study of the mechanical properties of radiation-grafted membranes was also critical (Figure 5 and Table 4). It was expected that their strength may have slightly decreased after grafting due to the wedging effect of the gel-type polyelectrolyte phase. However, the tensile strength of these membranes remained practically unchanged. In this case, it was reasonable that crosslinked membranes had a slightly higher tensile strength. Unexpectedly, the maximum elongation on the break of the radiation-grafted membranes was much more. If for MCG-0 the membrane elongation on break was approximately 3.6 times higher than for the initial PVDF film, after crosslinking, it decreased by one and a half times (Table 3). The reason for such changes seemed to be the reduced crystallinity of the membranes compared with the initial PVDF base film. Following the theoretical notions [58], the polymer molecules were in a coiled state, and the segmental motion leading to the straightening of the macromolecule was associated with overcoming the relatively small energy barrier of the internal rotation. This model assumed that the distance between the ends of an average chain varied in the same way as the sample size. Perhaps the more ordered stacking of PVDF molecules limited the folding of its molecules, while the grafting of the polystyrene lengthened this chain and increased its folding. At the same time, crosslinking limited the length of the unfolded chain and, consequently, the maximum elongation of the film (Figure 5 and Table 4). However, this was typically only when the applied force was sufficient to overcome the activation energy of the unfolding of the polymer chains. At the same time, at the first stage of stretching, the stress provided a small reversible change in the length of the sample, which was determined by the value of Young’s modulus (Figure 5). In this case, the increase in amorphous polymer fraction and the presence of backbones providing less ordered packing of polystyrene chains led to the facilitation of this stretching and a slight decrease in Young’s modulus (Table 4).

### 5.2. Testing of MEAs

**Figure 6:**
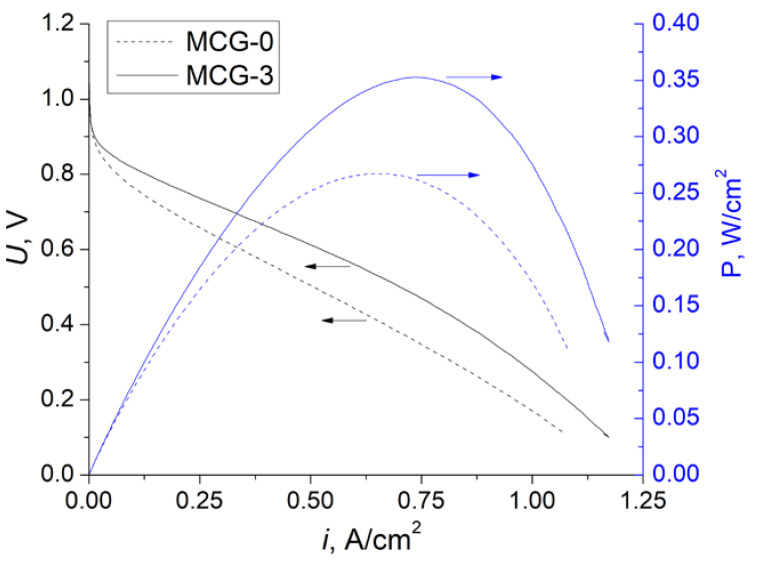
Voltammetric curves and dependences of the power density on the current density for MEAs with MCG-0 and MCG-3 membranes. The characteristics were measured at 65 °C and 100% gas RH. Arrows point to the respective Y-axes.

The high ionic conductivity and low gas permeability allowed us to hope for the high efficiency of the obtained membranes in PEMFC. Therefore, it is interesting to compare the PEMFC performance with the MCG-0 and MCG-3 membranes, the former having a higher conductivity and the latter having a lower gas permeability. It is worth noting that we used standard Nafion^®^-based catalytic ink without pressing when forming the MEAs. A comparison of the MEAs showed that the crosslinked membrane MCG-3 achieved 35% more peak power density than MCG-0 (Figure 6). In addition, the membrane MCG-3 provided half the H_2_-crossover compared with the membrane MCG-0, with 0.03 mA/cm^2^ versus 0.07 mA/cm^2^. Thus, in this case, one of the determining factors may be the lower gas permeability.

However, an equally important issue is the stability of the MEAs [59,60]. To evaluate this, we carried out accelerated stress tests. As the chemical degradation processes of membranes are electrocatalytic, the operating voltage determines the degradation rate. Therefore, the degradation of fuel cells was the most intense in open circuit conditions when the voltage reached its maximum values [61,62]. Accordingly, accelerated stress tests of the degradation processes were performed in the open circuit condition with hydrogen and oxygen supplied to the corresponding electrodes for an extended period. In addition, to accelerate the degradation, the cell temperature was increased to 90 °C, and the gas RH was reduced to 30%. The stability of the membranes under these conditions was evaluated by the open circuit voltage (OCV) change. Under similar conditions, the open-circuit voltage for the Nafion^®^-212 membrane dropped by 10% in 300 h [63].

**Figure 7:**
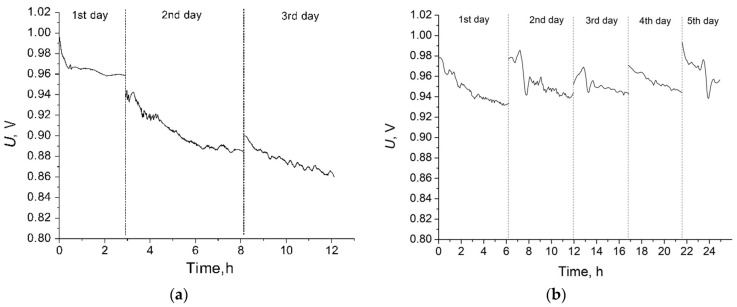
Open-circuit voltage changes in the MEAs with MCG-0 membrane (**a**) for three days and with the MCG-3 membrane (**b**) for five days. The cell temperature is 90 °C and the gas RH is 30%.

The change in the non-crosslinked MCG-0 membrane proceeded relatively quickly (Figure 7). During the first 20 min of operation, the OCV of the MEA with it decreased rapidly from 0.99 to 0.96 V and practically did not change during the first day of tests. During the second day of operation, the OCV dropped to 0.89 V, and during the third day to 0.87 V. It should be noted that during the night period when the gas supply was suspended, the MEA operation parameters were practically not resumed, i.e., the changes in the membrane were irreversible. Thus, we can conclude that the non-crosslinked membrane showed poor stability. On the other hand, tests of MEA with a crosslinked membrane MCG-3 showed quite different results (Figure 7). During each day of testing, the OCV of this MEA slowly decreased from 0.98 to 0.95 V. Moreover, during the night period, the MEA was restored.

After accelerated stress testing, high-frequency resistance was measured for both MEAs. The lower stability of the non-crosslinked membranes manifested itself in a more significant increase in area resistance compared with the crosslinked membrane from 116 to 440 Ohm∙cm^2^. In contrast, for the non-crosslinked sample, this parameter practically did not change and amounted to 130 and 140 Ohm∙cm^2^, respectively. The peak power density of MEA with MCG-3 did not change after 3-day stress testing, while in the case of MEA with MCG-0, this parameter decreased from 0.27 to 0.18 W/cm^2^ during the same testing time. In addition, after stress testing, an almost two-fold decrease in S_Pt_ for MEA with MCG-0 was observed (from 18 to 10 m^2^/g_Pt_), which may indicate poisoning of the cathode catalyst through membrane degradation products.

It is known that the degradation of membranes in PEMFC occurs as a result of the interaction between its various fragments with radicals formed through the interaction of hydrogen peroxide with transition metal cations M^x+^ Equations (1) and (2) coming from catalysts and other fuel cell components [64]:

M^2+^ + H_2_O_2_ → M^3+^ + •OH + OH^−^, (1)

M^3+^ + H_2_O_2_ → M^2+^ + •OOH + H^+^ (2)

Hydrogen peroxide formation can occur in two independent directions. One is the interaction of hydrogen and oxygen molecules diffusing through the membrane (crossover) on platinum particles passing into the membrane. However, the primary mechanism of its formation is the two-electron oxygen reduction reaction passing into the membrane [64]. The work of [65] showed that the membrane degradation rate increased significantly with the decreasing membrane thickness. This indicates that the rate of membrane degradation was determined mainly by its gas permeability.

**Figure 8:**
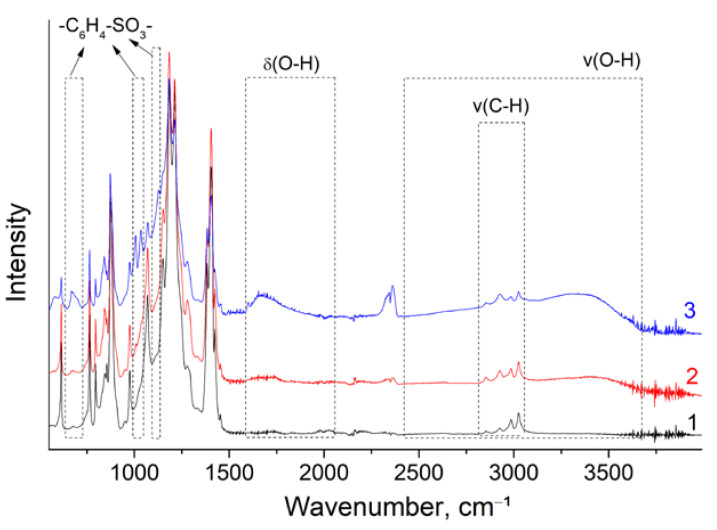
FTIR spectra of the initial PVDF film (1), and MCG-0 (2) and MCG-3 (3) membranes after the accelerated stress tests.

Comparing the properties of the obtained membranes and MEA crossover, it is evident that the gas permeability of the crosslinked MCG-3 was much lower. In addition, the diffusion rate of the formed radicals and hydrogen peroxide in the membrane could be limited by its crosslinking. These features are the reason for the significantly higher stability of the crosslinked material. It is also worth noting that crosslinking affected only the polystyrene sulfonate phase. The IR spectra of the membranes after the accelerated stress tests substantiated this (Figure 8). In the case of non-crosslinked membranes, the concentration of sulfonated polystyrene was much lower, which also explained the much higher resistance of MEA after the accelerated stress tests. This highlighted that if the degradation of the PVDF matrix occurred, this practically does not practically affect the PEMFC performance. This emphasizes that further ways to stabilize the membranes should be sought after, mainly for modifying the grafted polymer.

## 6. Conclusions

One of the promising trends in the search for new materials for PEMFC is radiation-grafted copolymers. In this work, the properties of membranes based on gamma-irradiated PVDF films and radiation-grafted sulfonated polystyrene with different degrees of crosslinking were synthesized. The grafting of polystyrene and its sulfonation is accompanied by the appearance of characteristic vibrations in the infrared spectra. The obtained materials possessed a proton conductivity up to 71 mS/cm at 30 °C and hydrogen permeability up to 5.2 cm^2^/s. According to these parameters, the obtained materials considerably exceeded Nafion^®^-212, which was chosen as a reference sample. This enabled the creation of a sufficiently high-power membrane electrode assembly (up to 0.4 W/cm^2^ at 65 °C). Using IR spectroscopy, we showed that the main cause of the MEA power drop during accelerated stress tests was the degradation of polystyrene sulfonate. The crosslinking of this polymer allowed for increasing the stability of the MEA essentially.

It has been shown that the radiation-grafted membrane retained the semi-crystalline structure of the original PVDF film. Still, the grafting reduced the degree of crystallinity due to the dilution effect and amorphization of some PVDF fragments.

## Figures and Tables

**Table 1:** Thickness (T), degree of grafting (DG), ion exchange capacity (IEC), hydration numbers (λ), hydrogen permeability (P(H_2_)), and conductivity (σ) of the membranes.

Membrane	T, μm	DG,%	IEC, mg-eq/g	λ,H_2_O/ SO_3_H	P(H_2_)∙10^8^, cm^2^/s	$\sigma_{H^{+}}^{30℃}$, mS/cm	$\sigma_{H^{+}}^{80℃}$, mS/cm
MCG-0	80	27	1.83	15	7.1	71	132
MCG-3	74	24	1.82	12	5.2	47	85
PVDF	50	0	-	-	0.2	-	-
Nafion^®^212 [45]	50.8	-	0.87	13.8	21.4	17	25

**Table 2:** Data regarding the quantitative analysis of the thermograms of the second heating cycle.

Sample	T_m1_, °C	T_m2_, °C	∆H_1_, J/g	∆H_2_, J/g	α_mem_ *, %	α_PVDF_, %
PVDF (initial film)	154–168	161–169	36.1	40.4	34.4	34.4
MCG-0	157–173	145–170	16.9	17.5	16.1	24.0
MCG-3	156–172	150–163	16.8	21.3	16.0	23.2

* calculated as the ratio of ∆H_1_ to the enthalpy of melting of 100% crystallized PVDF equal to 104.7 J/g [52].

**Table 3:** Proton conductivity of the obtained membranes (mS/cm) at 30 °C and the different relative humidity.

Membrane/RH	Contact with Water	95%	30%
MCG-0	71	18	0.42
MCG-3	47	25	0.44

**Table 4:** Values of Young’s modulus, yield strength, tensile strength, and elongation on break for the studied materials.

Sample	Young’s Modulus, %	Yield Strength, MPa	Tensile Strength at Break, MPa	Elongation on Break, %
MCG-0	1290 ± 60	34 ± 1	35 ± 1	107 ± 4
MCG-3	1317 ± 160	34 ± 1	39 ± 1	71 ± 14
PVDF	1460 ± 140	45 ± 1	37 ± 1	30 ± 10

## Data Availability

The data presented in this study are available on request from the corresponding author.

## Supplementary Materials

The following supporting information can be downloaded at: https://www.mdpi.com/article/10.3390/polym14183833/s1, Figure S1: The atomic concentration profile for MSC-0, Figure S2: The XRD pattern of initial PVDF film and MGC-3 membrane.
