# Supplementary material for: Membranes Based on Polyvinylidene Fluoride and Radiation-Grafted Sulfonated Polystyrene and Their Performance in Proton-Exchange Membrane Fuel Cells
Source: Polymers (Basel). 2022 Sep 14;14(18):3833. doi: 10.3390/polym14183833 (PMC9504926; doi:10.3390/polym14183833)
Supplement: Supplementary file 1 [file polymers-14-03833-s001.zip › polymers-1906186-supplementary.pdf]

Supplementary materials for article **"Membranes Based on Polyvinylidene Fluoride and Radiation-Grafted Sulfonated Polystyrene and Their Performance in Proton-Exchange Membrane Fuel Cells"**

Daniil V. Golubenko [1], Oleg V. Korchagin [2], Daria Yu. Voropaeva [1], Vera A. Bogdanovskaya [2] and Andrey B. Yaroslavtsev [1]

[1] Kurnakov Institute of General and Inorganic Chemistry RAS, 31 Leninsky Avenue, 119991, Moscow, Russian Federation
[2] Frumkin Institute of Physical Chemistry and Electrochemistry RAS, 31 Leninsky Avenue, 119071, Moscow, Russian Federation

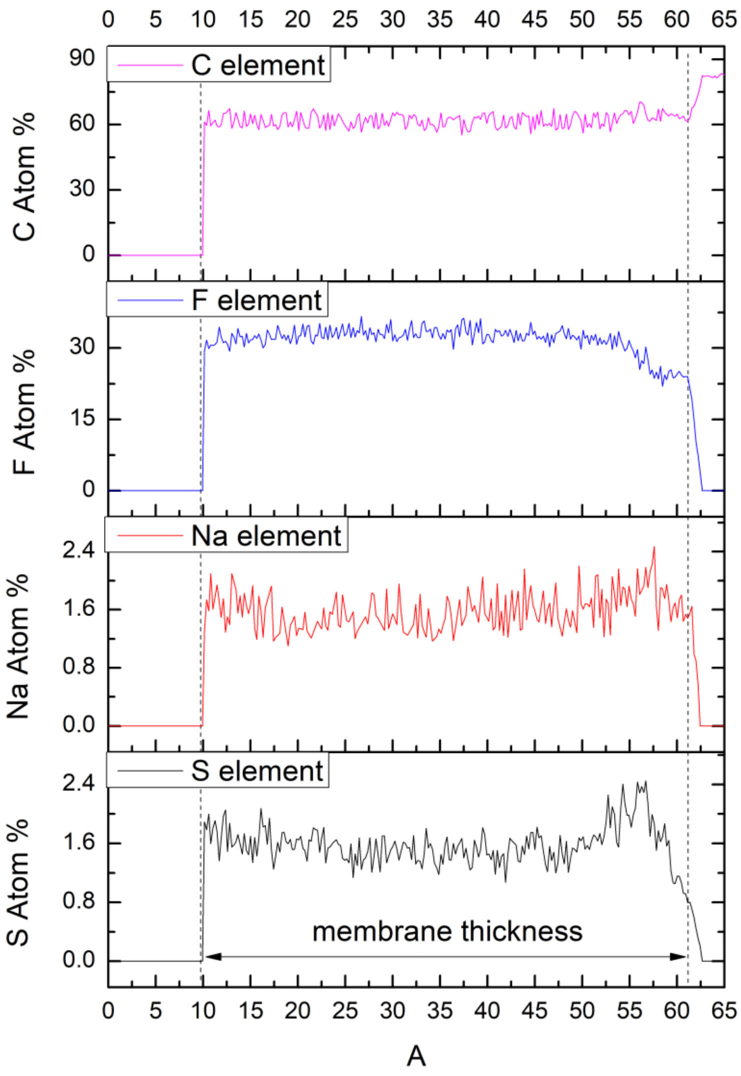

Figure S1. The atomic concentration profile for MSC-0.

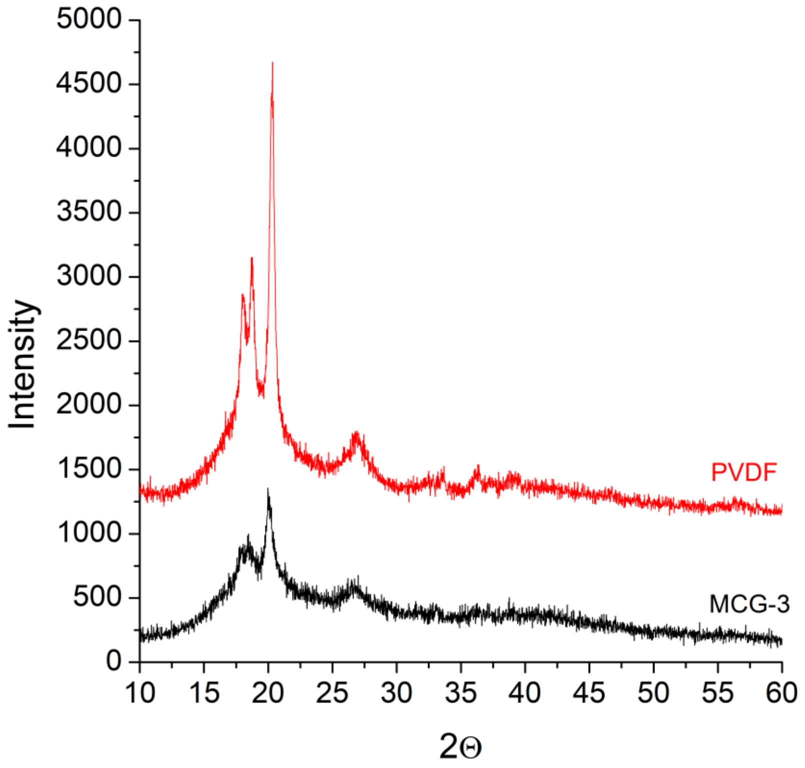

Figure S2. The XRD pattern of initial PVDF film and MGC-3 membrane.
